# Effectiveness of computer-navigated minimally invasive total hip surgery compared to conventional total hip arthroplasty: design of a randomized controlled trial

**DOI:** 10.1186/1471-2474-8-4

**Published:** 2007-01-11

**Authors:** Inge HF Reininga, Robert Wagenmakers, Inge van den Akker-Scheek, A Dennis Stant, Johan W Groothoff, Sjoerd K Bulstra, Wiebren Zijlstra, Martin Stevens

**Affiliations:** 1 Department of Orthopedics, University Medical Center Groningen, University of Groningen, PO Box 30001, 9700 RB Groningen, The Netherlands; 2 Medical Technology Assessment Office, University Medical Center Groningen, University of Groningen, The Netherlands; 3 Department of Health Sciences, University Medical Center Groningen, University of Groningen, The Netherlands; 4 Center for Human Movement Sciences, University Medical Center Groningen, University of Groningen, The Netherlands

## Abstract

**Background:**

Moderate to severe osteoarthrosis is the most common indication for Total Hip Arthroplasty (THA). Minimally Invasive Total Hip Surgery (MIS) and computer-navigated surgery were introduced several years ago. However, the literature lacks well-designed studies that provide evidence of superiority of computer-navigated MIS over a conventional THA technique. Hence, the purpose of this study is to compare (cost)effectiveness of computer-navigated MIS with a conventional technique for THA. It is our hypothesis that computer-navigated MIS will lead to a quicker recovery during the early postoperative period (3 months), and to an outcome at least as good 6 months postoperatively. We also hypothesize that computer-navigated MIS leads to fewer perioperative complications and better prosthesis positioning. Furthermore, cost advantages of computer-navigated MIS over conventional THA technique are expected.

**Methods/design:**

A cluster randomized controlled trial will be executed. Patients between the ages of 18 and 75 admitted for primary cementless unilateral THA will be included. Patients will be stratified using the Charnley classification. They will be randomly allocated to have computer-navigated MIS or conventional THA technique. Measurements take place preoperatively, perioperatively, and 6 weeks and 3 and 6 months postoperatively. Degree of limping (gait analysis), self-reported functional status and health-related quality of life (questionnaires) will be assessed preoperatively as well as postoperatively. Perioperative complications will be registered. Radiographic evaluation of prosthesis positioning will take place 6 weeks postoperatively. An evaluation of costs within and outside the healthcare sector will focus on differences in costs between computer-navigated MIS and conventional THA technique.

**Discussion:**

Based on studies performed so far, few objective data quantifying the risks and benefits of computer-navigated MIS are available. Therefore, this study has been designed to compare (cost) effectiveness of computer-navigated MIS with a conventional technique for THA. The results of this trial will be presented as soon as they become available.

## Background

Moderate to severe osteoarthrosis is the most common indication for total hip arthroplasty (THA). Incidence of THA in 2005 was 124 per 100,000 inhabitants (20,281 operations) in the Netherlands [1]. Over the past 40 years, THA has proven to be one of the most successful orthopedic interventions. The 15-year prosthesis survival rate exceeds 90% [2]. Thanks to excellent long-term results and technical improvements, more elderly people who were previously judged to be too old or too sick are considered suitable for THA. Moreover, the number of older adults is increasing [3]. Consequently, a boost will be seen in the demand for THA. Together with the shift toward greater cost effectiveness in healthcare, this growing demand triggers the introduction of potentially cost-saving procedures such as Minimally Invasive Total Hip Surgery (MIS).

MIS was introduced in the orthopedic community several years ago. Compared to the conventional incision technique for THA, a shorter incision is made. Proponents of MIS claim that it results perioperatively in less soft-tissue trauma (smaller skin incision and less muscle damage), reduced blood loss and fewer blood transfusion requirements. Postoperative benefits include less pain, quicker recovery (e.g earlier return to normal gait) and better cosmetic appearance [4-7]. Opponents of MIS argue that it leads to more complications, mainly due to poorer operative visualization of landmarks and vital structures [8]. Among the complications are neurovascular injury, femoral fracture and component malposition, which can result in more wear of the prosthesis. A higher risk for thromboembolism and infection is claimed, due to a longer operation time for MIS.

A solution to the poorer operative visualization is to consider using MIS in combination with computer navigation [7]. Several studies have shown that inaccuracies in prosthesis placement by means of conventional THA techniques can be significantly reduced by using computer navigation [9-11]. Some even hypothesize that MIS in combination with computer navigation will result in better positioning of the prosthesis, compared to conventional THA techniques [12].

In terms of cost effectiveness, MIS enthusiasts claim cost reduction due to earlier discharge from the hospital and sooner return to work, as MIS leads to a quicker recovery [13,14]. Opponents argue a cost increase as specialized equipment (e.g. computer navigation) is needed and operation time is longer [15].

Due to pressure from the industry as well as patients, the orthopedic community has widely embraced MIS. MIS and computer navigation are considered to be potential steps forward in the treatment of THA patients. The orthopedic literature however lacks well-designed studies that provide objective evidence on the effectiveness of computer-navigated MIS, especially in the early postoperative period (first 3 months), when its potential benefits are claimed to be substantial.

Hence, the purpose of this study is to conduct a randomized controlled trial to compare (cost) effectiveness of two THA techniques: computer-navigated MIS and a conventional technique. It is our hypothesis that computer-navigated MIS will lead to a quicker recovery during the early postoperative period (3 months), and to an outcome at least as good at 6 months postoperatively. We also hypothesize that computer-navigated MIS leads to fewer perioperative complications and better prosthesis positioning. From an economic perspective, cost benefits of computer-navigated MIS over conventional THA technique are expected. The present paper reports on the methodological design of the study.

## Methods/design

### Study design

A cluster randomized controlled trial will be conducted. Patients will be stratified into 3 groups based on the Charnley classification, by means of which total hip arthroplasty patients can be subdivided by degree of comorbidity affecting the function of walking. The Charnley classification recognizes three categories. Category A denotes patients with only one hip involved, in whom no other condition interferes with walking. Category B denotes patients with both hips involved but the rest of the body normal and therefore not responsible for any defect in walking. Category C denotes patients with some factors contributing to failure to achieve normal locomotion, such as polyarthritis or rheumatoid arthritis, or cardiovascular or respiratory disability [16]. By using this stratification, the influence of factors other than the THA affecting normal walking will be accounted for.

Within the strata, patients will be randomly allocated to have computer-navigated MIS or the conventional THA procedure by means of cluster randomization to avoid interaction between both patient groups. The random allocation sequence will be computer-generated by an independent planner of the Medical Assessment Office of University Medical Center Groningen (UMCG). The study design, procedures and informed consent are approved by the Medical Ethics Committee of UMCG.

### Study population

The study will be conducted at the Orthopedic Department of the UMCG. Patients between the ages of 18 and 75 who are admitted for primary cementless unilateral THA due to primary or secondary osteoarthrosis will be included. Patients with inflammatory polyarthritis or with a history of previous surgery on the affected hip will be excluded. Participation in the study is voluntary and patients have to provide informed consent before participation. The inclusion period is planned from March 2007 to May 2008.

### Intervention

#### Computer-navigated MIS

Patients in the MIS group will have surgery using the minimally invasive single-incision anterior approach [17]. The anterior approach is one of several possible approaches to the hip joint. Using special retractors, reamers and insertion handles it is possible to perform this procedure in a minimally invasive way, limiting the skin incision from about 15 cm. to about 8 cm. Advantage of the anterior approach is the possibility of using intermuscular spaces, avoiding muscle damage by cutting or detaching muscles and adding to the minimally invasive character of the approach.

An anterior incision centered over the hip joint is made in a supine patient. After division of skin and subcutis, the intermuscular space between the m. tensor fascia latae and the m. sartorius is identified and the overlying fascia is opened. The intermuscular plane between the m. tensor fascia latae and the m. sartorius is developed further down to the hip capsule. Subsequently the hip capsule is opened, allowing access to the hip joint. Preparation of the hip for implantation of a hip prosthesis can take place now, by in situ performance of osteotomy of the femoral neck, removal of the femoral head and reaming of the acetabulum, followed by insertion of an uncemented acetabular cup. After reaming of the femur an uncemented femoral component can be placed, followed by placement of a head on the femoral component, repositioning of the joint and closure in layers.

To optimize placement of the acetabular and femoral components of the total hip prosthesis, a computer navigation system (Stryker^® ^Navigation System iNstride Hip, Stryker Corporation, Kalamazoo, MI, U.S.A.) will be used. In order to use computer navigation it is necessary to place two trackers on the patient, which are used by the computer for referencing. These trackers are temporarily fixed on the patient by a small anchoring pin in the iliac crest and on the lateral side of the distal femur. These pins will cause no additional morbidity.

#### Conventional technique

Patients in the conventional technique group will have surgery using a standard posterolateral approach, in which the patient is placed in a lateral position. After transsection of the subcutis, the fascia latae and glutae are split. Next, the short external rotators are cut at the level of their insertion at the greater trochanter, so this approach is not muscle-sparing. After retraction of the short external rotators backwards, the hip capsule becomes visible and can be incised, allowing access to the hip joint. The rest of the operation will essentially take place in the same manner as the minimally invasive surgical technique.

In the computer-navigated MIS group as well as in the conventional technique group, the same femoral component (ABG II, Stryker Corporation) and acetabular cup (Trident^® ^Cup with X3 or Ceramic inlay, Stryker Corporation) will be used. The anesthetic, analgesic and postoperative physical therapy protocols will be standardized in both groups.

### Measurements

In this study, recovery is operationalized as the proportion of subjects with normal gait (no limping during walking) and as the self-reported functional status and health-related quality of life. Measurements will take place preoperatively (day of admission) and perioperatively, and 6 weeks and 3 and 6 months postoperatively. The amount of limping and self-reported functional status and health-related quality of life will be assessed preoperatively as well as postoperatively. Perioperative complications will be registered. Evaluation of prosthesis positioning will take place 6 weeks and 6 months postoperatively. The economic evaluation will focus on differences in costs between computer-navigated MIS and conventional THA technique. The evaluation will be performed from a societal perspective; costs within and outside the healthcare sector will be registered over a period of 6 months. Demographic data, diagnosis, height, weight and BMI, and ASA and Charnley classifications will also be recorded preoperatively.

#### Gait analysis

Functional status will be recorded objectively by means of gait analysis using body-fixed sensors. A major advantage of the body-fixed sensor-based approach is that these methods can be applied under real-life conditions; no expert laboratory is needed, and measurements can be made over longer periods of time and gait distances [18].

As walking is by far the most important aspect of functional status, the focus will lie on it – especially the extent of limping during walking (Duchenne limp), given that this is an evident indication of return to a normal gait. Gait parameters, such as accelerations and angular velocities of the upper trunk and pelvis, walking speed and step length, will be assessed while walking at slow, preferred and fast speeds, and while performing an additional attention-demanding task.

Limping can be measured by new methods and new hardware, both of which have been used in ongoing UMCG projects where gait function together with other measures are studied in patients after a hip or knee arthroplasty. Recent pilot work [19] has resulted in a new approach to assess compensatory movements of the trunk during walking. After the accuracy of this new method was confirmed by laboratory experiments, a field experiment showed that measures of pelvic and thoracic movements were related to (mean) walking speed, step length and step duration. The mean peak amplitudes in patients with and without Duchenne limp showed small but systematic differences [19]. It can be concluded that the new method is valuable for the assessment of compensatory trunk movements during gait. The approach allows for the simultaneous assessment of gait parameters and movements of the upper and lower trunk based on a combination of movement sensors.

#### Self-reported functional status and health-related quality of life

Self-reported functional status and health-related quality of life will be measured with questionnaires. The WOMAC will be used as a disease-specific outcome instrument to measure functional status. The SF-36 and EuroQol 5D are generic questionnaires and will be used to measure health-related quality of life. Patients' satisfaction with the results of the surgical procedure will be measured with the Patient Satisfaction Scale.

The Western Ontario and McMaster Universities Osteoarthritis Index (WOMAC) consists of three subscales measuring pain, stiffness and physical functioning. Patients have to score on a five-point Likert scale. The WOMAC has shown high validity and reliability [20]. The Dutch version of the WOMAC has also been considered valid and reliable [21]. The MOS 36-item Short Form Health Survey (SF-36) gives an indication of health-related quality of life, and is considered to be valid, reliable and reproducible [22]. The SF-36 is composed of 36 questions, organized into 8 multi-items scales: physical functioning, role limitations due to physical health problems, bodily pain, general health perceptions, vitality, social functioning, role limitations due to emotional problems and general mental health [22]. The WOMAC and SF-36 are the most widely used questionnaires in THA research [23,24]. The EuroQol 5D is a widely used and validated generic instrument that consists of 5 dimensions: mobility, self-care, usual activities, pain/discomfort and anxiety/depression [25]. The EuroQol 5D has to be seen as additional to the SF-36 and is embedded in this study protocol, as it is especially useful in combination with the economic evaluation that will be performed. The Patient Satisfaction Scale comprises 4 questions about satisfaction with pain relief, with improvement in function for home/yard work and with improvement in function for recreational activity, as well as overall satisfaction with surgery [26].

#### Perioperative measurements

Perioperatively, average surgical time, intraoperative blood loss, in-hospital transfusion rate and length of skin incision will be recorded.

#### Radiographic evaluation

At the Orthopedic Department of the UMCG a new digital measurement method has been developed with which postoperative measurements can be executed to objectify and quantify parameters of the quality of positioning of a total hip prosthesis [27]. The new procedure employs digital measurement techniques, which are far more reliable than conventional analogue techniques [28-30]. A radiographic evaluation of several parameters will take place by means of this new digital measurement method. Leg length differences, varus and valgus positioning of the stem, and inclination and anteversion of the acetabular component will be determined.

#### Economic evaluation

Outcomes of the above-mentioned measurements will be related to costs in additional economic analyses. These analyses will provide information on the probable cost effectiveness of computer-navigated MIS compared to conventional THA technique in the Dutch healthcare system.

Direct medical costs to be assessed include costs of computer-navigated MIS and conventional THA technique, blood transfusions, hospital admissions and costs related to length of hospital stay. In order to facilitate comparisons with other economic evaluations, unit prices (the price of one unit of each cost type included) will be based mainly on Dutch standard prices [31]. A questionnaire on medical costs outside the hospital (including physiotherapy, visits to general practitioners, nursing care and medication) and other (nonmedical) costs (e.g. absence of work) will be administered to the patients.

### Sample size

It is our hypothesis that computer-navigated MIS will lead to better recovery during the early postoperative period (3 months), and at least as good at 6 months postoperatively. In order to detect a difference of 0.254 in the proportion of subjects with normal gait after 3 months of follow-up with 80% power at a significance level of 0.05 in a one-sided test of a difference between two proportions, two groups of 50 subjects are required. With an expected dropout rate of 10%, a total of 110 patients is needed. At 6 months, the effect of MIS and conventional THA technique on gait (limping) will be compared in a non-inferiority setting. To establish non-inferiority, the lower bound of the 95% confidence interval of the difference between the two treatment groups will be compared with a non-inferiority margin delta. If the whole confidence interval of the difference between the two treatments is smaller than the non-inferiority margin delta, non-inferiority is established. The non-inferiority margin delta is chosen in this study at a value of 0.10, indicating that a difference in proportion of subjects with a normal gait of 0.10 is considered clinically equivalent. To deduce non-inferiority with 80% power at a significance level of 0.05 with expected proportions of subjects with normal gait of 0.95, using a non-inferiority margin delta of 0.10, two groups of 60 subjects are required. With an expected dropout rate of 10%, a total of 132 patients is needed.

### Statistical analysis

All statistical analyses will be computed using the Statistical Package for the Social Sciences (SPSS, Inc., Version 12.0, 2003, Chicago). Descriptive statistics will be used to describe both research groups. Analysis of variance (ANOVA) and chi-square procedures will be used to evaluate between-group differences at baseline. Random effect models will be applied for longitudinal analyses. In order to enable statistical conclusions on differences in (skewed distribution of) costs between groups during the study, nonparametric confidence intervals will be constructed based on results of bootstrap analyses. For all test procedures, a probability value of less than 0.05 will be considered as statistically significant.

## Discussion

Over the last few years, MIS has become a widely used technique for THA. Several authors [5,6,32] have concluded that MIS is a safe and reproducible procedure. Chimento et al. [6] executed a randomized prospective study comparing MIS to a standard approach. There was no difference between the groups in number of patients being able to achieve rehabilitation milestones. However, patients who underwent MIS demonstrated decreased blood loss and limped less 6 weeks postoperatively, indicating a quicker return to a more normal gait. An objective gait analysis was not performed though. In a retrospective cohort study, Woolson et al. [33] attempted to determine whether there was a difference in surgical parameters and component positioning of MIS compared to a standard THA technique. The results showed no differences with respect to surgical parameters. It was concluded that there were no benefits associated with MIS except for a smaller scar. However, more malpositions of the acetabular and femoral components were seen in the MIS group. Malpositioning is a potential complication of MIS due to poorer operative visualization. Computer navigation can be a preventive tool as it permits accurate orientation and fixation of the prosthesis without the need for visualization of bony landmarks. Computer navigation has proved to decrease inaccuracies in prosthesis placement by means of conventional THA technique [9-11].

Wixson and MacDonald [12] found more reproducible acetabular component placement in a series of computer-navigated MIS as compared to a cohort of a conventional THA technique. They concluded that using MIS in combination with computer navigation can improve the accuracy of component placement. DiGioia and colleagues [7] compared MIS and conventional surgery, both with the help of computer navigation. At 3 months, MIS patients had significantly better results in limping and stair-climbing, and at 6 months in limping, walking and stair-climbing as determined with the Harris hip score. They used a matched control group and patients were not randomized.

The literature lacks well-designed studies that provide objective evidence to conclude that computer-navigated MIS is superior to a conventional procedure for THA. Additionally, there are conflicting reports on the cost advantages of (computer-navigated) MIS over conventional THA techniques [13-15].

Purpose of the study presented in this article is to compare (cost) effectiveness of two THA techniques: computer-navigated MIS and a conventional technique. Since computer-navigated MIS is less invasive to muscles and skin, advantages are expected in the early postoperative phase in terms of a quicker recovery. We also hypothesize that it leads to fewer perioperative complications, better prosthesis positioning and cost savings. The results of this study will be presented as soon as they become available.

## Competing interests

The author(s) declare that they have no competing interests.

## Authors' contributions

MS and RW originated the idea for the study, led on its design, and will supervise the project. JWG and SKB were co-applicants on the successful funding proposal. MS, RW, WZ, JWG, SKB, ADS and IAS participated in the design of the study and in developing the research protocols. ADS provided statistical consultation. IHFR will coordinate the trial and is responsible for data acquisition. All authors read and corrected draft versions of the manuscript and approved the final manuscript.

## Pre-publication history

The pre-publication history for this paper can be accessed here:



## References

[B1] Prismant Hospital Statistics. http://www.prismant.nl/informatieproducten/ziekenhuisstatistieken.

[B2] Havelin LI, Engesaeter LB, Espehaug B, Furnes O, Lie SA, Vollset SE (2000). The Norwegian Arthroplasty Register: 11 years and 73,000 arthroplasties. Acta Orthop Scand.

[B3] Ostendorf M, Johnell O, Malchau H, Dhert WJ, Schrijvers AJ, Verbout AJ (2002). The epidemiology of total hip replacement in The Netherlands and Sweden: present status and future needs. Acta Orthop Scand.

[B4] Berry DJ, Berger RA, Callaghan JJ, Dorr LD, Duwelius PJ, Hartzband MA, Lieberman JR, Mears DC (2003). Minimally invasive total hip arthroplasty. Development, early results, and a critical analysis. Presented at the Annual Meeting of the American Orthopaedic Association, Charleston, South Carolina, USA, June 14, 2003. J Bone Joint Surg Am.

[B5] Howell JR, Masri BA, Duncan CP (2004). Minimally invasive versus standard incision anterolateral hip replacement: a comparative study. Orthop Clin North Am.

[B6] Chimento GF, Pavone V, Sharrock N, Kahn B, Cahill J, Sculco TP (2005). Minimally invasive total hip arthroplasty: a prospective randomized study. J Arthroplasty.

[B7] DiGioia AM, Plakseychuk AY, Levison TJ, Jaramaz B (2003). Mini-incision technique for total hip arthroplasty with navigation. J Arthroplasty.

[B8] Callaghan J (2006). Skeptical perspectives on minimally invasive total hip arthroplasty. J Bone Joint Surg Am.

[B9] DiGioia AM, Jaramaz B, Blackwell M, Simon DA, Morgan F, Moody JE, Nikou C, Colgan BD, Aston CA, Labarca RS, Kischell E, Kanade T (1998). The Otto Aufranc Award. Image guided navigation system to measure intraoperatively acetabular implant alignment. Clin Orthop Relat Res.

[B10] Kalteis T, Handel M, Bathis H, Perlick L, Tingart M, Grifka J (2006). Imageless navigation for insertion of the acetabular component in total hip arthroplasty: is it as accurate as CT-based navigation?. J Bone Joint Surg Br.

[B11] Leenders T, Vandevelde D, Mahieu G, Nuyts R (2002). Reduction in variability of acetabular cup abduction using computer assisted surgery: a prospective and randomized study. Comput Aided Surg.

[B12] Wixson RL, MacDonald MA (2005). Total hip arthroplasty through a minimal posterior approach using imageless computer-assisted hip navigation. J Arthroplasty.

[B13] Bertin KC (2005). Minimally invasive outpatient total hip arthroplasty: a financial analysis. Clin Orthop Relat Res.

[B14] Duwelius PJ (2006). Two-Incision Minimally Invasive Total Hip Arthroplasty: Techniques and Results to Date. Instr Course Lect.

[B15] Sikorski JM, Chauhan S (2003). Computer-assisted orthopaedic surgery: do we need CAOS?. J Bone Joint Surg Br.

[B16] Charnley J (2005). The long-term results of low-friction arthroplasty of the hip performed as a primary intervention. Clin Orthop Relat Res.

[B17] Rachbauer F, Hozack J, Krismer M, Nogler M, Bonutti PM, Rachbauer F, Schaffer JL, Donnelly WJ (2004). Minimally invasive single incision anterior approach for total hip arthroplasty – early results. Minimally invasive total joint arthroplasty.

[B18] van den Bogert A, Read L, Nigg BM (1996). A method for inverse dynamic analysis using accelerometry. J Biomech.

[B19] Zijlstra A (2005). The sensitivity of a method with body fixed sensors to determine trunk orientation in patients with hip osteoarthritis. MSc thesis.

[B20] Soderman P, Malchau H (2001). Is the Harris hip score system useful to study the outcome of total hip replacement?. Clin Orthop Relat Res.

[B21] Roorda LD, Jones CA, Waltz M, Lankhorst GJ, Bouter LM, van der Eijken JW, Willems WJ, Heyligers IC, Voaklander DC, Kelly KD, Suarez-Almazor ME (2004). Satisfactory cross cultural equivalence of the Dutch WOMAC in patients with hip osteoarthritis waiting for arthroplasty. Ann Rheum Dis.

[B22] Aaronson NK, Muller M, Cohen PD, Essink-Bot ML, Fekkes M, Sanderman R, Sprangers MA, te Velde A, Verrips E (1998). Translation, validation, and norming of the Dutch language version of the SF-36 Health Survey in community and chronic disease populations. J Clin Epidemiol.

[B23] Ethgen O, Vanparijs P, Delhalle S, Rosant S, Bruyere O, Reginster JY (2004). Social support and health-related quality of life in hip and knee osteoarthritis. Qual Life Res.

[B24] Lingard E, Hashimoto H, Sledge C (2000). Development of outcome research for total joint arthroplasty. J Orthop Sci.

[B25] Brooks R (1996). EuroQol: the current state of play. Health Policy.

[B26] Mahomed NN, Sledge CB, Daltroy LH, Fossel AH, Katz JN (2006). Self-administered satisfaction scale for joint replacement arthroplasty. J Bone Joint Surg Br.

[B27] The B, Diercks RL, Stewart RE, van Ooijen PM, van Horn JR (2005). Digital correction of magnification in pelvic x rays for preoperative planning of hip joint replacements: theoretical development and clinical results of a new protocol. Med Phys.

[B28] Ebramzadeh E, Sangiorgio SN, Lattuada F, Kang JS, Chiesa R, McKellop HA, Dorr LD (2003). Accuracy of measurement of polyethylene wear with use of radiographs of total hip replacements. J Bone Joint Surg Am.

[B29] Ilchmann T, Mjoberg B, Wingstrand H (1995). Measurement accuracy in acetabular cup wear. Three retrospective methods compared with Roentgen stereophotogrammetry. J Arthroplasty.

[B30] Ilchmann T (1997). Radiographic assessment of cup migration and wear after hip replacement. Acta Orthop Scand Suppl.

[B31] Oostenbrink JB, Al MJ, Rutten-van Molken MP (2003). Methods to analyse cost data of patients who withdraw in a clinical trial setting. Pharmacoeconomics.

[B32] Berger RA (2003). Total hip arthroplasty using the minimally invasive two-incision approach. Clin Orthop Relat Res.

[B33] Woolson ST, Mow CS, Syquia JF, Lannin JV, Schurman DJ (2004). Comparison of primary total hip replacements performed with a standard incision or a mini-incision. J Bone Joint Surg Am.

